# Health risk assessment of heavy metals in soils and food crops from a coexist area of heavily industrialized and intensively cropping in the Chengdu Plain, Sichuan, China

**DOI:** 10.3389/fchem.2022.988587

**Published:** 2022-09-01

**Authors:** Qing Liu, Xiaohui Li, Lei He

**Affiliations:** ^1^ School of Chemistry and Chemical Engineering, Anshun University, Anshun, China; ^2^ College of Life Science, Sichuan Normal University, Chengdu, China

**Keywords:** heavy metals, food crops, health risk, daily intake, Chengdu Plain

## Abstract

Environmental pollution caused by rapid industrial activities are becoming increasingly drastic, particularly its impact on soil and plant health. The present study was conducted to investigate the heavy metal (loid) (As, Cd, Cu, Hg, Pb, and Zn) concentrations in soils and food biomass crops and estimate the potential health risks of metals to humans *via* consumption of contaminated food biomass crops from Shifang, a periurban agricultural areas in the Chengdu Plain, Sichuan, China. Results revealed that the soils have been experiencing a substantial accumulation of heavy metals, especially for Cd, with a mean of 0.84 mg kg^−1^, about six times higher than the background values, of which 98% exceeded the pollution warning threshold of the China Soil Environmental Quality Standards. A total of 78% of all the grain part failed the national food standard for Cd. No significantly positive relationships between metal levels in food biomass crops and in the corresponding soils, indicated metals enrichment in soils were not entirely reflected to crops contaminant burdens. Estimated daily intake (EDI) of all the metals except for Pb, exceeded the oral reference dose (RfD) or the minimal risk levels recommended by USEPA and ATSDR. Target hazard quotients (THQs) of all the metals except for Cd was less than one indicated that potential health risk to the local inhabitant originated mainly from Cd exposure *via* cereals consumption. Mitigation strategies to curtail Cd-contaminated soils and crops Cd burdens need careful tailoring to meet the needs of health and safety in this region.

## 1 Introduction

Nowadays, environmental pollution and the greenhouse effect are becoming increasingly drastic. Among them, potential environmental and human risks of exposure to heavy metals through diet become an important issue of public health concern, but such information remains still fragmentary and scattered in an area of intensively cropping and heavily industrialized coexist (Masindi and Muedi, 2018; Briffa et al., 2020). Heavy metals are ubiquitous in the environment, with either natural or anthropogenic origin (Wu et al., 2016). Anthropogenic activities, such as mining, solid waste disposal, sludge applications, and industrial processing are the main sources of heavy metals soil contamination (Sodango et al., 2018; Timothy and Tagui Williams, 2019; Sharma et al., 2021). In addition, excessive use of pesticides and fertilizers, and wastewater irrigation also play an important role in the contamination of foodstuffs by heavy metals (Loutfy et al., 2012; Zwolak et al., 2019; Qin et al., 2021). Toxic heavy metals released by anthropogenic activities into ecosystems may lead to geo-accumulation, bio-accumulation, and biomagnification. In agricultural ecosystems, excessive accumulation of heavy metals in agricultural soils leads to elevated heavy metals uptake by food biomass crops, which is of great concern because of potential health risk to humans (Ali et al., 2019; Afonne and Ifediba, 2020; Hasan et al., 2020). Consumption of food biomass crops contaminated with heavy metals is a major food chain route for human exposure. Recently, there have been increasing interests in human health risk caused by consuming heavy metal contaminated food (Sall et al., 2020; Zheng et al., 2020; Ahmad et al., 2021; Alengebawy et al., 2021). The Food and Agricultural Organization of the United Nations (FAO), World Health Organization (WHO), United States Environmental Protection Agency (USEPA), the Agency for Toxic Substances and Disease Registry (ATSDR), and other regulatory bodies of various countries have established maximum permissible limits (MPL) of heavy metals in foodstuffs and offered some methods for health risk assessment. Based on these methods, numerous studies have been conducted on potential health risk assessment of heavy metals contaminated in soils and crops in different regions (Saha et al., 2016; Ishtiaq et al., 2018; Bello et al., 2019; Lien et al., 2021; Setia et al., 2021; Udom et al., 2022). But such information from an intensively cultivated areas remains still fragmentary (Bhatti et al., 2018; Zheng et al., 2020).

The Chengdu Plain, the“Land of Abundance” in China, is an important agricultural region and has also experiencing rapid change of socio-economic structur e changes. Urbanization, industrialization, and agricultural intensification have caused an increase of large amounts of metal-contained agrochemicals, wastes, and sewages in the agricultural environment. Previous studies in the Chengdu Plain reveal an obvious increase of heavy metals, especially cadmium (Cd) and mercury (Hg) in soils in the past decades (Qin et al., 2013; Wang et al., 2017; Deng et al., 2019; Wang et al., 2019). Increased heavy metals in soil results not only in soil quality deteriorating but may also affect agricultural product safety. There is a probable accumulation of heavy metals in crops grown in this region. However, studies have suggested that the knowledge of total concentration of metals alone is not sufficient to evaluate phytotoxic risk and human health risk (Yuswir et al., 2015; Tapia-Gatica et al., 2020); Huang et al. (2018) suggest that exposure to heavy metals through rice intake was the most important single health risk contributor. Dietary intake through contaminated foods has become the main route of heavy metal intake by humans (Chen et al., 2018). Therefore, the risk assessment of exposure to heavy metals through diet becomes an important health issue.

The coexist area of heavily industrialized and intensively cropping occupied a considerable proportion in the Chengdu Plain. Many previous studies only considered the levels of heavy metals in the soils and/or vegetables (Liu et al., 2004; Jin et al., 2008; Qin et al., 2013) and no studies have investigated the bio-accumulation of heavy metals in food crops from the soils in the coexist area. On the one hand, there are a large number of industrial pollution sources from such as the chemical industry, metal smelting, and cement production in the coexist areas, pollutants enter into farmland are inevitable through atmospheric deposition, wastewater, and solid waste discharge. On the other hand, highly intensive farming systems also bring a certain amount of heavy metals to the agricultural environment through the application of chemical fertilizers and pesticides. Health consequences of these pollution to local residents need to understand assessment, which has certain implications for changes in local planting structures and risk mitigation strategies or the safe usage of farmlands.

In order to enable the development of appropriate environmental and/or health guidelines, it is essential to have an understanding of the universal range of heavy metals concentrations in crops on the intensively cultivated area. Such data are also important to assist in assessing any potential risk to the environment or human health. The purpose of this study is to 1) identify the concentration of heavy metals in soils, rice, and maize; 2) evaluate the potential health risk associated with heavy metals through consumption of rice and maize using estimated daily intake (EDI) and target hazard quotient (THQ).

## 2 Materials and methods

### 2.1 Study area

**FIGURE 1:**
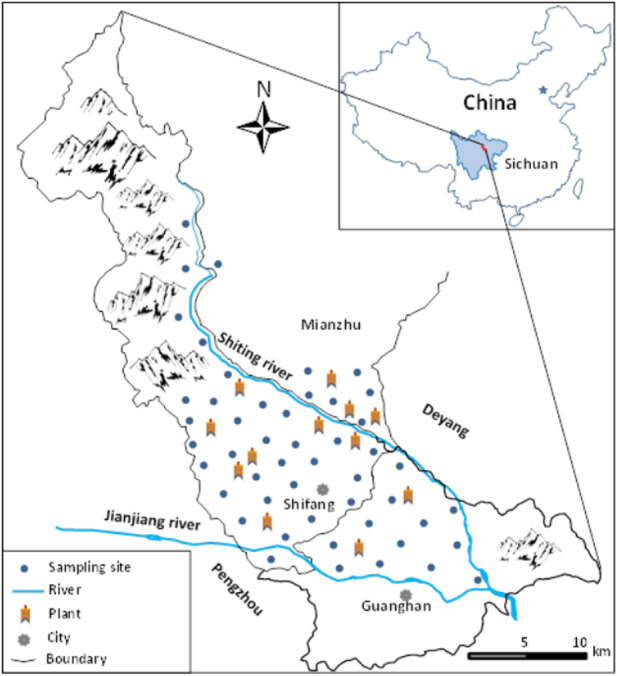
Schematic map of sampling sites in the study area, Sichuan province (southwest China).

The research area is composed of the whole flat area of Shifang city and parts of Guanghan city and Mianzhu city, situating at the northwest of the Chengdu Plain, Sichuan province, southwestern China (103°58′–104°22′E, 30°58′–31°23′N) (Figure 1). Climate is subtropical humid climate, with mean annual temperature of −16°C and mean annual rainfall of 940 mm. Parent materials of soils are mainly alluvial of the Min River. Major soil types are Hapli-Stagnic Anthrosols and Gleyi-Stagnic Anthrosols in the Chinese Soil Taxonomy (Gong and Li, 2001).As a heavily industrial activities and intensively farming coexist area, the study area has diverse industrial clusters, including food, metallurgical, construction materials, pharmaceutical, chemical, and leather industry in the flat part, whereas the mountainous/hill part being phosphorus ore, coal, and limestone mining.

Rice is cultivated on 90% of the total arable lands, rest be used for maize, wheat, vegetable, and mushroom cultivating (Wang M. et al., 2016). Agriculture depends on agrochemicals. It is a typical area that intensive crop farming mingled with heavily industrial operations in the Chengdu Plain.

### 2.2 Sample collection

The systematic random sampling method was used to collect samples. A sampling grid overlay on the study region (Figure 1), then the samples were collected at the node areas of a regular grid of about 3 km × 3 km. At each sampling site, a clean plastic shovel was used to collect 5–7 randomly subsamples at the 0–20 cm depth to form one representative composite sample of at least 500 g by the quartering method and put in a cleaned zip-lock plastic bag for the laboratory analysis. At each soil sampling sites, 3–5 subsamples of the edible part of maize mature seeds and/or the ear of rice were collected at random, and a composite sample at least 300 g was made for each crop. In total, the samples of 40 paddy soils, 10 dryland soils, 40 rice, and 10 maize were collected.

### 2.3 Analyses of samples

Soil samples were screened of debris and stones, air-dried, and crushed to pass through a 2-mm sieve. Each sample was homogeneized and quartered, representative subsamples of ≤2 mm size fraction were grounded in an agate mortar to pass a 0.149-mm sieve and prepared for chemical analysis. Samples of rice and maize were washed with deionized water to remove all visible soil particles or dusts, oven-dried at 60°C. After rice husks were removed, rice grains were grounded in a stainless steel mill to a fine powder and stored in plastic bags for further chemical analysis. Corn kernels were also prepared in the same way as analytical samples.

Soil pH was measured by a pH meter with soil/H_2_O ratio of 1:2.5 (soil:solution, dry w/v). The organic matter content was determined by the Walkley-Black procedure. Cation exchange capacity (CEC) was determined using NH_4_OAc at pH 7.0, the leaching method of the Soil Survey Staff (1996). Soil samples were digested by concentrated acid mixture (HNO_3_, HClO_4_, and HF), and food crop samples were digested with HNO_3_ and HClO_4_ in a 5:1 ratio. The acid digested soil and crop samples were filtered and diluted with distilled water to 50 and 10 ml, respectively. Concentration of total Pb, Cd, Cu, and Zn in the digests was measured using an atomic absorption spectrophotometer (Analyst 800 P.E.) equipped with a heated graphite furnace system (THGA-800 P.E.), while As and Hg were determined by atomic fluorescence spectrometer (AFS-830a).

### 2.4 Quality control and assurance

To ensure the quality of metals analysis, certified reference material (CRM) (from the National Research Center for Standards in China, Beijing) including Sichuan basin soil (GBW07428) and Sichuan rice flour (GBW10044) was used to validate the analysis. The average rice flour CRM recoveries ranged from 91 to 101%, 94 to 100%, 96 to 101%, 98 to 101%, 96 to 103%, and 90 to 105% for Cd, As, Pb, Cu, Zn, and Hg, respectively. The mean recoveries for soil CRM’s ranged from 89 to 100%, 97 to 104%, 98 to 102%, 97 to 100%, 98 to 101%, and 95 to 103% for Cd, As, Pb, Cu, Zn, and Hg.

### 2.5 Data analysis

#### 2.5.1 Bio-accumulation factor

Metal concentrations of soils and grains were calculated on the basis of dry weight. The bio-accumulation factor (BAF), a ratio of the contaminant in food crops to the concentration in the soil substrate, was calculated using the following equation:

$$\text{BAF}=\frac{C_{plant}}{C_{soil}},$$

where *C*_*plant*_ and *C*_*soil*_ represent the heavy metal (loid) concentration in the edible part of food crops and soils on dry weight basis, respectively.

#### 2.5.2 Estimated daily intake of metals

The estimated daily intake (EDI) of the specific metal depended on both the metal concentration in the edible part of food crops and the amount of consumption of the respective food. The EDI was determined by the following equation:

$$\text{EDI}=\frac{C_{metal}\times CF\times W_{food}}{BW},$$

where *C*_*metal*_ (mg kg^−1^, on dry weight basis) is the concentration of metals in contaminated crops; *CF* denotes the conversion factor, the *C*_*metal*_ of both rice and maize were converted with a factor of 0.86 because home-stored rice and maize commonly contain water under 14% (w/w); *W*_*food*_ represents the daily average consumption of food crops in the study area; and *BW* is the average body weight. According to the dietary intake surveyed by Zhu et al. (2000), the local inhabitants had an average consumption per person (average 65 kg in body weight) of 363 and 45 g/day for rice and maize, respectively, for children (average 30 kg in body weight), estimated intake account for about 60% of consumption for adults.

#### 2.5.3 Target hazard quotient

Health risks for locals through the consumption of contaminated rice and maize was assessed based on the target hazard quotient (THQ). The THQ is a ratio of determined dose of a pollutant to a reference dose level. If the ratio is less than one, the exposed population is assumed to be safe (USEPA, 2012); THQ is described by the following equation:

$$\text{THQ}=\frac{EF\times ED\times FIR\times C}{RfD\times BW\times TA}\times 10^{-3},$$

where EF is the exposure frequency (365 days/year); ED is the exposure duration (70 years), equivalent to the average lifetime; FIR is the food ingestion rate (for adults, rice: 363 g/person/day, maize: 45 g/person/day; for children, rice: 218 g/person/day and maize: 27 g/person/day) (Zhu et al., 2000); C is the metal (loid) concentration in food (μg g^−1^); RfD is the oral reference dose (As = 0.3 μg kg^−1^ d^−1^, Hg = 0.16 μg kg^−1^ d^−1^, Cd = 1 μg kg^−1^ d^−1^, Pb = 4 μg kg^−1^ d^−1^, Cu = 10 μg kg^−1^ d^−1^, Zn = 300 μg kg^−1^ d^−1^) (USEPA, 2008; USEPA, 2013; ATSDR, 2013); BW is the average body weight (65 kg), and TA is the averaging exposure time for non-carcinogens (365 days/year ×ED).

#### 2.5.4 Statistical analysis

Data were statistically analyzed using a statistical package SPSS 20. Shapiro–Wilk test is used to determine whether sample data have been drawn from a normally distributed population. When the assumption of normality was met, the mean was selected to test the statistical significance of the data, including comparison the mean of two and multiple groups and analysis of variance (ANOVA), with a significance level of *p* < 0.05, and the figures also presented with the mean values and standard errors. When the assumption of normality was violated, the median was selected to do Mann–Whitney test for two groups and Kruskal–Wallis test for multiple groups, and Spearman’s correlation analysis was used to test the correlation assumption.

## 3 Results

### 3.1 Heavy metals in soils

Basic soil characteristics and the concentrations of As, Cd, Hg, Pb, Cu, and Zn in soils are presented in Table 1. Soil is generally slightly acidic (mean pH 6.48), with a range of acidic (pH 4.54) to slightly alkaline (pH 7.99), in which pH value of 36 soil samples was less than seven, accounting for 72%. Soil organic matter (SOM) ranged between 54 and 112 g kg^−1^, with a mean of 101 g kg^−1^. Cation exchange capacity (CEC) varied considerably from 2.35 to 21.16 cmol kg^−1^, a difference of approximately nine times. The pH value in the rainfed lands that maize cultivated soils were not markedly different, comparing with the paddy fields that rice cultivated soils (*p* = 0.919). There was also no obvious difference in SOM between rainfed lands and paddy fields (*p* = 0.422). CEC in the paddy land was significantly lower than that in rainfed lands (*p* = 0.049).

**TABLE 1:** Characteristics and metal levels of the soils collected from the study area (matters content on dry weight basis).

Property	Range	Mean (*n* =50)	SE^a^	Background value^b^	SEPA RSV^c^ (pH = 6.5–7.5)
pH (H_2_O)	4.54–7.99	6.48	0.85	—	—
SOM(g kg^−1^)	54.04–112.23	101.04	20.31	—	—
CEC (cmol kg^−1^)	2.35–21.16	7.04	0.45	-	-
As (mg kg^−1^)	3.81–33.18	8.01	0.75	3.77	30
Cd (mg kg^−1^)	0.51–1.90	0.84	0.04	0.14	0.30
Hg (mg kg^−1^)	0.12–0.33	0.18	0.03	0.14	0.30
Pb (mg kg^−1^)	8.05–80.33	24.49	1.69	20.70	250
Cu (mg kg^−1^)	16.66–70.57	25.72	1.42	23.01	50
Zn (mg kg^−1^)	53.58–159.59	80.21	3.19	65.12	200

a SE denote standard error.

b Background value from Yao (1987).

c Risk-based screening values (RSV) of soil environmental quality risk control standard for soil contamination of agricultural land (GB 15618-2018) (Ministry of Ecology and Environment of the PRC, 2018).

**FIGURE 2:**
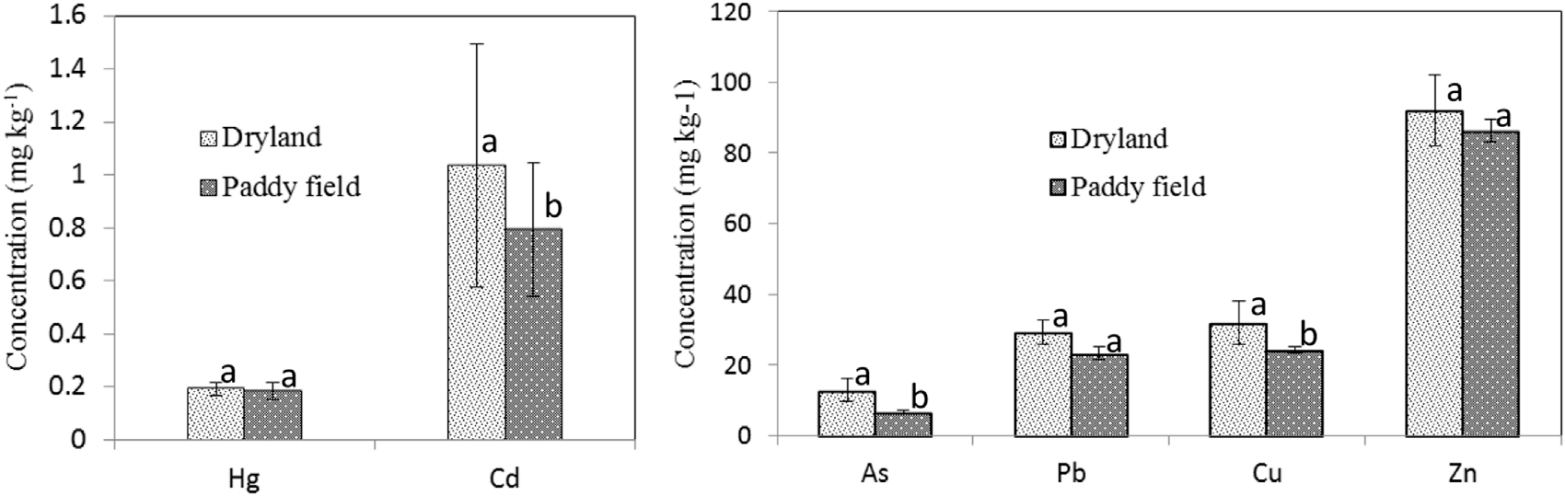
Metal concentrations (dry weight basis) in the soils from different types of farming in the study area. Data are mean ± 1SE, *n* = 40 for paddy soils and *n* = 10 for dryland soils. For each element, means with the same letter are not significantly (*p* > 0.05) different.

Mean concentrations of As, Cd, Hg, Pb, Cu, and Zn in soils were 8.01, 0.84, 0.19, 24.49, 25.72, and 80.12 mg kg^−1^, respectively (Table 1). Except for Cd, all other metals were below the risk control standard for soil contamination of agricultural land (Ministry of Ecology and Environment of the PRC, 2018) (Table 1). A considerable buildup for all the metals in the soils were observed when comparison with the background values, indicating that soils in the study area had a considerably contaminated by heavy metals, especially Cd and As. Concentration of metals was higher in rainfed soil than in paddy soil (Figure 2), with As (*p* = 0.001), Cd (*p* = 0.027), and Cu (*p* = 0.028) significantly higher in rainfed soil than paddy soil, and the rest [Pb (*p* = 0.156), Hg (*p* = 0.174), Zn (*p* = 0.47)] insignificantly different.

### 3.2 Heavy metals in food crops

The average concentrations and ranges of heavy metals (on dry weight basis) in the edible portions of food crops grown in the investigated soils are given in Table 2. The average concentrations of Cd in rice and maize were 0.46 and 0.26 mg kg^−1^, respectively (Table 2), 78% of crop samples exceeded the MPL for Cd of Chinese standard (Ministry of Health of the PRC, 2017). Mean content of Pb in rice and maize was 0.30 and 0.29 mg kg^−1^, respectively, also overtaking the MPL. The results indicated that both rice and maize in the study area exhibited a conspicuous Cd and Pb pollution. But As, Hg, Cu, and Zn concentrations were substantially lower than the MPL in rice and maize grown in the soils of the research area (Table 2). All the metal contents except for Cu (*p* = 0.001) and Cd (*p* =0.05) are insignificantly different between rice and maize, but the trends of heavy metal accumulations, in general, was in the order of rice > maize (Table 2).

**TABLE 2:** Metals concentrations (on dry weight basis) in the edible parts of food crops collected from the study area.

Metal	Rice (*n* =40)		Maize (*n* =10)		
	Range	Mean ± SE	Range	Mean ± SE	MLs^a^
As (mg kg^−1^)	0.04–0.17	0.07 ± 0.01	0.04–0.13	0.06 ± 0.01	0.15 (0.2)^b^
Cd (mg kg^−1^)	0.09–1.78	0.46 ± 0.06	0.05–0.77	0.26 ± 0.08	0.2 (0.1)
Hg (mg kg^−1^)	0.002–0.21	0.01 ± 0.01	0.002–0.02	0.01 ± 0.01	0.02
Pb (mg kg^−1^)	0.06–0.58	0.30 ± 0.02	0.02–0.50	0.29 ± 0.05	0.2
Cu (mg kg^−1^)	1.48–6.33	4.14 ± 0.17	1.21–5.30	2.73 ± 0.48	10
Zn (mg kg^−1^)	27.49–53.54	39.55 ± 0.83	35.58–49.13	41.72 ± 1.48	50

a Maximum levels of contaminants in foods (GB 2762-2017) (Ministry of Health of the PRC, 2017).

b Number in parenthesis indicate maximum levels of metals in food grains other than rice.

### 3.3 Heavy metal transfer from soil to food crop

**FIGURE 3:**
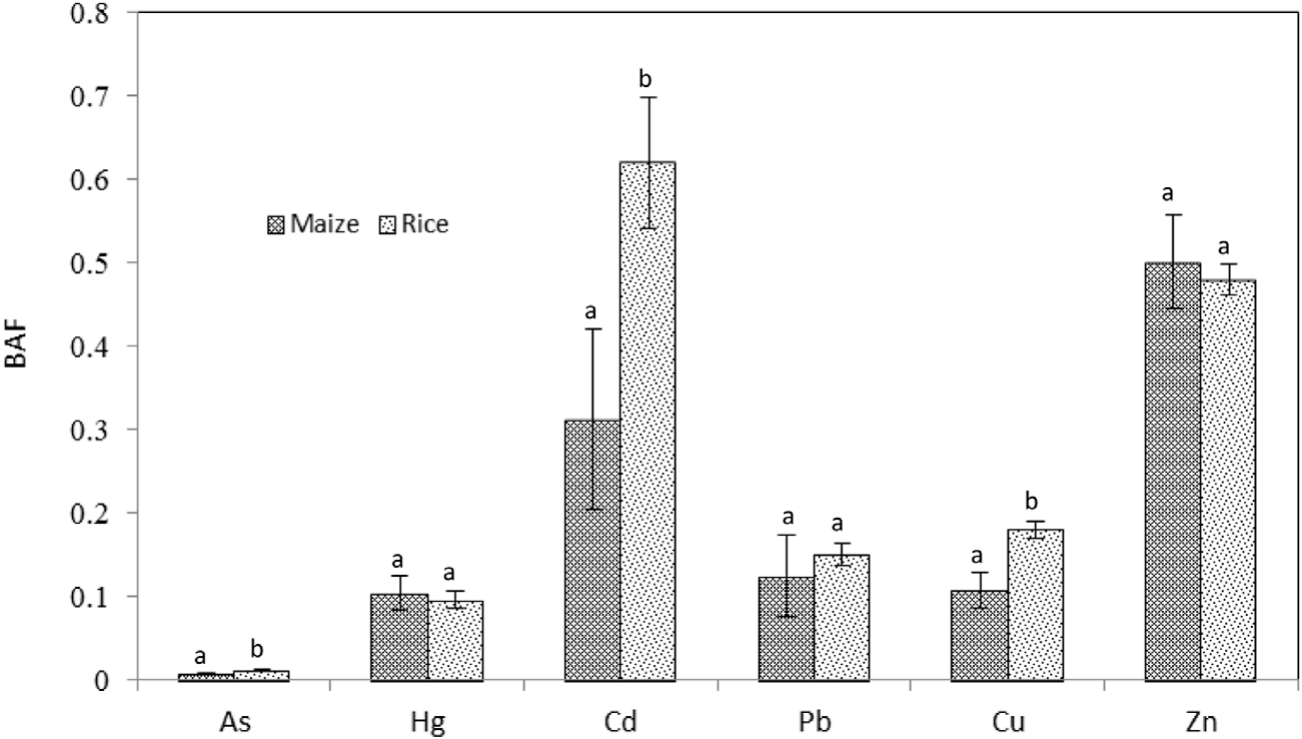
Bio-accumulation factors (BAF) for different metals, a ratio of heavy metals concentration in the edible part of maize and rice to that in the corresponding soil at the study area. Data are mean ± 1SE. For each element, means with the same letter are not significantly (*p* > 0.05) different.

The bio-accumulation factor (BAF) for heavy metal transferring from soils to food crops tended to be in the order of Cd > Zn > Cu > Pb > Hg > As, and there was significantly difference in BAF values among metals (*p* < 0.001) (Figure 3). As, Cd, and Cu in the edible parts of rice were markedly higher than that of maize (Figure 3, all *p* < 0.05), whereas Hg, Pb, and Zn buildup in the corresponding parts of crops had no obvious difference. Correlation analysis reveals no significant correlation between metal concentrations in soils and in the edible parts of the plants except for Cu (*p* = 0.026), indicating that the metals content in plant does not fully reflect the total metal level in soils.

### 3.4 Daily intake of metals through food consumption and human health risks

Daily intake of heavy metal was estimated based on the average food consumption in the study area. The estimated daily intake (EDI) *via* consumption of rice and maize for adults and children is given in Table 3. EDI of Hg, As, Pb, Cd, Cu, and Zn for adults was 0.11, 0.44, 1.62, 2.43, 21.52, and 214.83, respectively, whereas for children it was 0.31, 0.55, 2.11, 3.12, 38.02, and 379.52, respectively. According to the oral reference dose (RfD) recommended by USEPA, ASTDR and Cal EPA, EDI of As, Cd, and Cu for adults had exceeded the reference dose (Table 3), while EDI, except for that of Pb, for children, surpassed the recommended limit. Moreover, EDIs through the consumption of rice were significantly higher than through the consumption of maize because the dietary habits of local inhabitant are centered on rice. In particular, EDI values to the local children tended to be higher over adults, indicating that the children had a relatively significant health risks *via* the consumption of metals contaminated foods.

**TABLE 3:** Estimated daily intake (EDI) of metals by consumption of rice and maize at the investigation area (the EDI values based on the body weight of 65 and 30kg for the adults and children, respectively).

Groups	Type of food	DI^a^ (g d^−1^)	As	Hg	Cd	Pb	Cu	Zn
			µg kg^−1^ d^−1^					
Adults	Rice	363	0.43	0.11	2.21	1.43	19.91	190.02
	Maize	45	0.04	0.01	0.22	0.21	1.62	24.81
	Total		**0.47** ^e^	0.11	**2.43**	1.62	**21.52**	214.83
Children	Rice	218	0.51	0.33	2.92	1.91	25.92	247.22
	Maize	27	0.05	0.01	0.21	0.23	2.13	32.33
	Total		**0.55**	**0.31**	**3.12**	2.11	**38.02**	**379.52**
RfD^b^			0.3	0.16^c^	1	4	10^d^	300

a DI represents dietary intake (Zhu et al., 2000).

b Oral reference dose base on USEPA (2013).

c Oral reference exposure level (REL) recommended by OEHHA at CalEPA (2013).

d Minimal risk levels (MRLs) recommended by Agency for Toxic Substances and Disease Registry ATSDR (2013).

e Data in bold represents exceeding the RfD recommended by USEPA, indicating a potential health risk.

**FIGURE 4:**
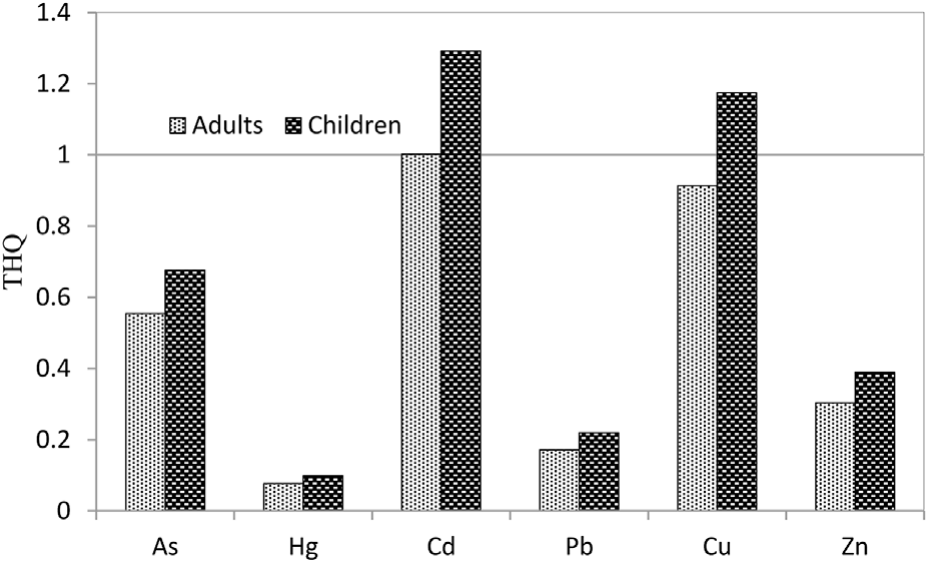
THQ values of metals through consumption of rice and maize grown at the sampling sites of the study area (a histogram above the reference line may be subjected to potentially higher health risk).

Target hazard quotient (THQ) of metals through the ingestion of rice and maize for adults and children is shown in Figure 4. While THQ of As, Hg, Pb, Zn, and Cu for adults was below one, indicating health risk was low, THQ of Cd was close to one, suggesting a potential health threat. Analogously, THQ of As, Hg, Pb, and Zn for children from consumption of rice and maize was below one, which suggested that health risk was insignificant. Conversely, THQ of Cd and Cu was bigger than one, indicating that health risk these two metals was of a concern.

## 4 Discussion

### 4.1 Heavy metals in soils and crops

Increasing evidence (Ren et al., 2006; Tang et al., 2007; Jin et al., 2008; Qin et al., 2013) indicate that high Cd concentration in paddy soil and rice in the Chengdu Plain is a problem. All the metal concentrations except for Cd, although were still below the Grade II of the EQSS (SEPA, 2018), a substantial accumulation of metals in the soils was found when compared with the background values (Yao, 1987), on average concentrations of As, Hg, Pb, Cu, and Zn increasing up to 112, 30, 18, 12, and 23%, respectively (Table 1). These results agreed with the findings of previous studies in the Chengdu Plain (Qin et al., 2013; Wang et al., 2017; Deng et al., 2019). Cd concentrations in 49 soils were above the pollution warning threshold of the EQSS, accounting for about 98%, indicating that soils suffered generally from Cd contamination. This may have been due to the fact that diversity industries embedded in an intensively cultivated area, such as phosphorus chemicals, leather chemicals, old or currently active mining or ore processing facilities, with mine waste runoff or overspill tainted irrigation water, atmospheric deposition resulting from ore smelting, and application of agrochemicals, all may contribute to Cd contamination and others metals buildup in soils (Qin et al., 2013). Many previous studies suggested that long-term wastewater irrigation led to elevated levels of heavy metals in soils (Elbana et al., 2013; Christou et al., 2014; Meng et al., 2016; Abuzaid and Fadl, 2018) and revealed that the heavy metals content in soils were markedly influenced by stationary sources such as non-ferrous metal smelter, coal-fired power plant (Reza et al., 2015; Yang et al., 2017; Semenov et al., 2019; Wang et al., 2020), and non-point sources as use of fertilizers, pesticides, and bactericides (Ouyang et al., 2016; Zhang et al., 2021). These activities are inevitable in an intensively farming and heavily industrial activities coexist area.

Concentrations of Cd, As, and Cu in rainfed soils are significantly different from that in paddy soils (Figure 2), suggesting that different farming styles may potentially impact on metal concentrations in soil. It is a fact that rainfed lands are commonly used to cultivate vegetables, with a high ratio of rotation and increasing the input of agrochemicals, and more potential metals of anthropic sources being added to rainfed soils in compare to paddy soils. Metal elements may have different behaviors such as bioavailability, leachability, and mobility in various environments. An extractable form by DTPA is commonly applied to evaluate availability in previous many studies (Kim et al., 2015; Zahedifar et al., 2017; Kaninga et al., 2020). It was reported that Cd and Cu were readily extracted by DTPA compared to Pb and Zn (Singh et al., 1998), suggesting that Cd and Cu have more bioavailability in the same field condition, and therefore it should be possible to incur more leachability and/or mobility in a wet–dry cropping rotation due to the function of water. The mobile behavior of As in soils was affected by many factors such as pH and amorphous Al and Fe contents (Violante et al., 2010; Álvarez-Ayuso et al., 2016), especially, reduction condition possibly facilitates As releasing from soil because of reducing As (Ⅴ) to As (Ⅲ) (Pigna et al., 2015; Guénet et al., 2017). These behaviors would show why that As levels was different between rainfed soils and paddy soils.

Heavy metal accumulation in crops is a serious concern due to potential public health implications. The data from present study indicated that the average concentration of Cd in rice and maize was 2.3 and 2.6-fold higher than the MPL (Table 2), respectively. Similarly, Pb contents in rice and maize also surpassed the MPL by about1.5 times (Table 2). The concentration of Cd in rice from the Chengdu Plain was in balance with the findings of previous studies in a mining-affected area of Hunan province (Du et al., 2013; Chen et al., 2016; Wang X. et al., 2016) but lower than those in the Dabaoshan mine area in Guangdong province (Zhuang et al., 2009). While the Cd level in corn grains was beyond that of the corn grown at Qingchengzi Pb/Zn mine soil in Liaoning province (Li et al., 2014). On average buildup of Pb in the edible part of both rice and maize reached up to about 0.30 mg kg^−1^, ranging from 0.06 to 0.58 mg kg^−1^ for rice and 0.02–0.50 mg kg^−1^ for maize (Table 2), which was less than those in mining-affected areas (Zhuang et al., 2009). Analysis showed that no strong positive relationship between metals in the soils and in the crops. Conversely, a negative relationship between soil metal contents and crops for Cu, in addition to Cd were observed. Such inverse relationships were also reported by Khan et al. (2008) for vegetables. This may suggest that knowing total metal levels in soils use to assess health risk is inappropriate.

Soil-to-plant transfer is one of key pathways of human exposure to metals through food chain (Loutfy et al., 2006). Our results showed that BAF differ significantly among metals (*p* < 0.001) or between crops (Figure 3). Seemingly, Cd, As, and Cu transfer from soil to rice were easier than to maize (all *p* < 0.05), but the rest of the metals did not like such trend. However, the Cd, As, and Cu levels in rainfed soils where maize was cultivated, on the whole, are higher than those in paddy soils where rice was cultivated (Figure 2), suggesting that the accumulation effect depends not only on the crop’s physiological properties but also on mobility and availability of metals in soils, it does not appear to be entirely associated with the total element concentrations in the soils. Some studies found that leafy vegetables can generally accumulate Pb and Cd to a higher extent than non-leafy vegetables (Zhuang et al., 2009; Nabulo et al., 2010; Chang et al., 2014; Gebeyehu and Bayissa, 2020). Cd is usually considered a highly mobile heavy metal in regard to moving from soil-to-plant and is of primary concern in soil and food contamination, particularly in rice cropping systems (Kim et al., 2015; Zhao et al., 2015). A high average BAF for Cd in rice correspond to a lower mean content of total Cd in paddy soils (Figures 2, 3). Gu et al. (2018) investigated the BAF of rice for Cd, Cu, Pb, and Zn, the results indicated that Cd and Zn showed stronger bio-accumulation and mobility capability. These findings demonstrate that Cd accumulation in rice is mainly influenced by its availability, rather than total amount in soils, which support the conclusions of many previous studies (Du et al., 2013; Jing et al., 2020).

### 4.2 Health risk assessment

An important aspect in assessing risk to human health from potentially harmful chemicals in food is the knowledge of the dietary intake of such substances. Based on average concentration of metals in the edible part of each food crops and the respective consumption rate (Zhu et al., 2000), EDIs of As, Cd, and Cu by consumption of rice and corn grains for the local adults were 0.44, 0.24, and 21.52 μg kg^−1^ d^−1^, and for the local children were 0.55, 0.31, and 38.02 μg kg^−1^ d^−1^, respectively (Table 3). These EDIs are far below those in the mining-affected areas (Zhuang et al., 2009; Du et al., 2013), but exceed the oral reference dose (RfD) recommended by USEPA (2013) and ATSDR (2013). Analogously, EDIs of Hg and Pb for the local children exceed also the RfD limits, but not for adults (Table 3). The EDIs through consumption of rice were significantly higher than that of maize due to rice as staple crop of local inhabitant. Thus, adverse health effects induced by ingesting contaminated food crops arise largely from rice consumption. Moreover, the local children intakes of metals by consumption of contaminated food crops was about 1.25–2.8 times higher than those of the local adults due to children consumption 1.3 times more food than adults relative to their body weight (Table 3). A similar phenomenon was also reported by Kim et al. (2013), they found that the mean intakes of Cd at ages 1–2 were the highest in different age groups of Korea through the intake of various agricultural products grown in greenhouse. Ding et al. (2018) investigated trace elements in soils and selected agricultural plants in the Tongling mining area of China, their findings revealed that EDI of the trace elements, except Cd, were generally below the maximum tolerable daily intake. These estimates were also consistent with the long-term dietary intake assessment on other contaminants performed by the FAO/WHO (FAO/WHO, 2005). Therefore, children as a susceptible group have a higher health risk through consuming the same contaminated foodstuff than adults.

The estimation of target hazard quotient (THQ) offers an indication of the risk level due to pollutant exposure (USEPA, 2017; Shaheen et al., 2016; Miranzadeh Mahabadi et al., 2020). Estimated THQs of As, Hg, Pb, Zn, and Cu through consumption of rice and maize were below one for adults, while this value for Cd approached one. Concerning children, THQs of As, Hg, Pb, and Zn were also less than one, but for Cd and Cu were beyond one, suggesting that Cd and Cu exposure through daily intakes of rice and corn grains locally produced have posed a severe health risk to the local residents (Figure 4), in agreement with the conclusion of Jin et al. (2009). Although the ingested dose of heavy metals from cereals is not equal to the absorbed pollutant dose in reality due to a fraction of intake heavy metals being excreted (Balkhair and Ashraf, 2016; Yaradua et al., 2020), if considering dietary intakes from the locally produced non-cereal foods consumption such as vegetables, meat, eggs, and milk, THQ of metals, especially Cd, is certainly higher and the health risk even more severe. Consequently, effective mitigation measures are necessary to cure Cd-contaminated soils and to reduce the metal transferring from soil to crops in this region.

## 5 Conclusion

The soils from a heavily industrialized and intensively cultivated area in the Chengdu Plain have been experiencing a considerable accumulation of heavy metals comparison with the background values. These enrichments are not entirely reflected to crops metals burdens due to difference in bioavailability and/or mobility among metals and/or in physiological properties between crops. Health risk identified by the estimated daily intake (EDI) and target hazard quotients (THQ) suggest that potential health risk to the local inhabitant is mainly from Cd exposure, resulting from rice consumption. Mitigation strategies to curtail Cd-contaminated soils and crops Cd burdens need careful tailoring to meet the needs of health and safety in this region in the future.

## Data Availability

The original contributions presented in the study are included in the article/Supplementary Material; further inquiries can be directed to the corresponding author.
